# Supplementary material for: Biomechanical analyses of pterygotid sea scorpion chelicerae uncover predatory specialisation within eurypterids
Source: PeerJ. 2022 Dec 9;10:e14515. doi: 10.7717/peerj.14515 (PMC9745958; doi:10.7717/peerj.14515)
Supplement: Supplemental Information 1 [file peerj-10-14515-s001.zip › peerj-77894-Supplementary_Tables_10-13 StaffRL.docx]

| Taxon |  |
| --- | --- |
| Molluscs |  |
|  | *Cyclora valvatiformis* |
|  | *Cyclora imbricata* |
| Ostracods |  |
|  | *Primitia mundula* |
|  | *Primitia scaphoides* |
| Arthropleurids |  |
|  | *Eoarthropleura devonica* |
| Millipedes |  |
|  | *Gaspestria genselorum* |
| Eurypterids |  |
|  | *Parahughmilleria* sp. |
|  | ***Pterygotus anglicus*** |
|  | *Pterygotus* sp. |
| Scorpions |  |
|  | Mesoscorpion indet |
| Ostracoderms |  |
|  | *Yvonaspis campbelltonensis* |
|  | *Yvonaspis jexi* |
|  | *Yvonaspis westolli* |
| Placoderms |  |
|  | *Phlyctaenius acadicus* |
|  | *Phlyctaenius atholi* |
|  | *Phlyctaenius stenosus* |
| Acanthodians |  |
|  | *Ankylacanthus incurvus* |
|  | *Cheiracanthus? costellatus* |
|  | *Gyracanthus incurvus* |
|  | *Homacanthus gracilis* |
|  | *Mesacanthus semistriatus* |
| Chondrichthyans |  |
|  | *Climatius latispinosus* |
|  | *Doliodus problematicus* |
|  | *Protodus jexi* |

**Supplemental Table 10**: Faunal list of the Lower Devonian (Emsian) Campbellton Formation, Canada. Modelled species in bold. Data derived from Shear et al. (1996), Wilson (2006), Kennedy et al. (2012), Miller et al. (2012), Fyffe et al. (2016), and Burrow et al. (2017).

| Taxon |  | Formation (s) |
| --- | --- | --- |
| Bryozoans |  |  |
|  | *Ptilodictya* sp. | Patrick Burn |
| Tentaculitids |  |  |
|  | *Tentaculites ornatus* | Patrick Burn |
| Annelids |  |  |
|  | *Turrilepas wrighti* | Patrick Burn |
| Brachiopods |  |  |
|  | *Atrypa reticularis* | Patrick Burn |
|  | *Dicoelosia* (=*Orthis*) *biloba* | Patrick Burn |
|  | *Lingula minima* | Kip Burn |
|  | *Leptaena* (=*Strophomena*) *rhomboidalis* | Patrick Burn |
|  | *Platyschisma* sp. | Kip Burn |
|  | *Resserella* (=*Orthis*) *elegantula* | Patrick Burn |
| Molluscs |  |  |
|  | *Acroculia* sp*.* | Patrick Burn |
|  | *Ctenodonta thracioides* | Patrick Burn |
|  | *Orthoceras annulatum* | Patrick Burn |
|  | *Orthoceras ludense* | Patrick Burn |
| Ostracods |  |  |
|  | *Entomozoe tuberosa* | Patrick Burn |
| Phyllocarids |  |  |
|  | *Ceratiocaris* *papilio* | Kip Burn, Patrick Burn |
|  | *Ceratiocaris stygia* | Kip Burn |
| Thylacocephalans |  |  |
|  | *Ainiktozoon loganense* | Patrick Burn |
| Trilobites |  |  |
|  | *Calymene blumenbachii* | Patrick Burn |
|  | *Encrinurus punctatus* | Patrick Burn |
|  | *Phacops stokesi* | Patrick Burn |
|  | *Podowrinella staitonensis* | Patrick Burn |
|  | *Staurocephalus murchisoni* | Patrick Burn |
| Chasmataspids |  |  |
|  | *Loganamaraspis dunlopi* | Patrick Burn |
| Synziphosurines |  |  |
|  | *Cyamocephalus loganensis* | Patrick Burn |
|  | *Pseudoniscus* sp. | Patrick Burn |
| Eurypterids |  |  |
|  | ***Erettopterus bilobus*** | Kip Burn, Patrick Burn |
|  | *Hardieopterus lanarkensis* | Patrick Burn |
|  | *Hughmilleria* sp. | Kip Burn, Patrick Burn |
|  | *Nanahughmilleria lanceolata* | Patrick Burn |
|  | *Parahughmilleria* cf*. hefteri* | Kip Burn |
|  | *Slimonia acuminata* | Patrick Burn |
| Agnathans |  |  |
|  | *Birkenia* sp. | Kip Burn |
|  | *Jamoytius kerwoodi* | Patrick Burn |
|  | *Loganellia scotica* | Patrick Burn |

**Supplemental Table 11:** Faunal list of the Silurian (latest Llandovery and Wenlock) Patrick and Kip Burn formations, Scotland. Modelled species in bold. Data derived from Jones & Woodward (1885), Rolfe & Fritz (1966), Howells (1982), Boucot & Janis (1983), Briggs & Clarkson (1987), Märss & Ritchie (1997), Anderson (1999), Tetlie & Braddy (2003), Tetlie & Poschmann (2008), and Perrier et al. (2019).

| Taxon |  |
| --- | --- |
| Molluscs |  |
|  | *Archaeopraga pinnaeformis* |
|  | *Boionautilus bohemicus* |
|  | *Cardiolinka bohemica* |
|  | *Corbuloceras corbulaturn* |
|  | *Drahomira barrandei* |
|  | *Drahomira glaserae* |
|  | *Drahomira rugata* |
|  | *Kopaninoceras fluminese* |
|  | *Orthonychia* cf*. anguis* |
|  | *Parakionoceras originále* |
|  | *Parakionoceras magister* |
|  | *Parakionoceras docens* |
| Brachiopods |  |
|  | *Bleshidium triste* |
|  | *Gracianella graciosa* |
|  | *Opatrilkiella minuta* |
|  | *Opsiconidion simplex* |
| Ostracods |  |
|  | *Boucia ornatissima* |
|  | *Ceratiocaris bohemica* |
|  | *Vltavina bohemica* |
| Trilobites |  |
|  | *Prionopeltis striata* |
|  | *Scharyia nympha* |
| Eurypterids |  |
|  | ***Acutiramus bohemicus*** |
|  | *Acutiramus nobilis* |
|  | *Eusarcana acrocephala* |
|  | *Paracarcinosoma accrocephala* |
|  | *Slimonia acuminala* |
| Acanthodians |  |
|  | *Machaeracanthus* sp*.* |

**Supplementary Table 12**: Faunal list of the Silurian (Pridoli) Požáry Formation, Czech Republic. Modelled species in bold. Data derived from Chlupáč et al. (1980), Kříž et al. (1986), Kříž (1992), Chlupáč (1994), Mergl (2001, 2019), Horný (2004, 2005), Südkamp & Burrow (2007), Manda & Turek (2009), Manda & Frýda (2010), and Budil et al. (2014).

| Taxon |  | Formation (s) |
| --- | --- | --- |
| Annelids |  |  |
|  | *Spirorbis* sp. | Nellenköpfchen |
| Echinoderms |  |  |
|  | *Luxaster martini* | Klerf |
| Brachiopods |  |  |
|  | "*Lingula*" *cornea* | Nellenköpfchen |
|  | *Mutationella* sp*.* | Klerf |
|  | *Mutationella* cf. *confluentina* | Nellenköpfchen |
|  | *Retzia* sp. | Klerf |
| Molluscs |  |  |
|  | *Goniophora* aff*. schwerdi* | Nellenköpfchen |
|  | *Limoptera* (*Klinoptera*) *rhenana* | Nellenköpfchen |
|  | *Leiopteria* (*Leiopteria*) *crenatolamellosa* | Nellenköpfchen |
|  | *Leptodomus* cf. *barroisi* | Nellenköpfchen |
|  | *Modiola antiqua* | Nellenköpfchen |
|  | *Modiolopsis ekpempusa* | Klerf |
|  | *Modiolopsis* sp. | Nellenköpfchen |
|  | *Palaeoneilo beushauseni* | Nellenköpfchen |
| Ostracods |  |  |
|  | *Poloniella* cf*. confluens* | Klerf |
|  | *Rebskeella waxweilerensis* | Klerf |
| Trilobites |  |  |
|  | *Burmeisterella delattrei* | Klerf |
| Chasmataspids |  |  |
|  | *Diploaspis casteri* | Nellenköpfchen |
| Synziphosurines |  |  |
|  | Synziphosurine indet. | Klerf |
|  | *Willwerathia laticeps* | Klerf, Nellenköpfchen |
| Arachnids |  |  |
|  | *Alkenia mirabilis* | Nellenköpfchen |
|  | *Archaeomartus levis* | Nellenköpfchen |
|  | *Archaeomartus tuberculatus* | Nellenköpfchen |
|  | *Archaeometa devonica* | Nellenköpfchen |
|  | *Waeringoscorpio hefteri* | Nellenköpfchen |
|  | *Xenarachne willwerathensis* | Klerf |
| Eurypterids |  |  |
|  | *Adelophthalmus sievertsi* | Klerf |
|  | *Alkenopterus brevitelson* | Nellenköpfchen |
|  | *Carcinosoma* sp. | Nellenköpfchen |
|  | *Drepanopterus struvei* | Nellenköpfchen |
|  | *Erieopterus* sp. | Klerf |
|  | ***Jaekelopterus rhenaniae*** | Klerf, Nellenköpfchen |
|  | *Moselopterus ancylotelson* | Nellenköpfchen |
|  | *Moselopterus elongatus* | Nellenköpfchen |
|  | *Parahughmilleria hefteri* | Klerf, Nellenköpfchen |
|  | *Parahughmilleria major* | Nellenköpfchen |
|  | *Pruemopterus salgadoi* | Klerf |
|  | *Rhenopterus diensti* | Klerf |
|  | *Rhenopterus macrotuberculatus* | Nellenköpfchen |
| Ostracoderms |  |  |
|  | *Drepanaspis* sp. | Nellenköpfchen |
| Placoderms |  |  |
|  | *Aggeraspis heintzi* | Nellenköpfchen |
|  | *Herasmius* sp. | Klerf |
|  | *Kujdanowiaspis* sp. | Nellenköpfchen |
|  | *Lunaspis broilii* | Nellenköpfchen |
|  | *Phlyctaenius pusilla* | Nellenköpfchen |
|  | *Prosphymaspis constricra* | Nellenköpfchen |
|  | *Tiaraspis* sp. | Klerf |
|  | *Tiaraspis subtilis* | Nellenköpfchen |
| Agnathans |  |  |
|  | Pteraspid indet. | Klerf |
|  | *Rhinopteraspis dunensis* | Nellenköpfchen |
| Porolepiforms |  |  |
|  | *Durialepis edentatus* | Klerf |

**Supplemental Table 13**: Faunal list of the Lower Devonian (Emsian) Klerf and Nellenköpfchen formations, Germany. Modelled species in bold. Data derived from Fürsich & Hurst (1980), Dunlop & Poschmann (1997), Anderson et al. (1998), Wehrmann et al. (2005); Poschmann (2006), Poschmann & Franke (2006), Poschmann & Tetlie (2006), Braddy et al. (2008), Poschmann et al. (2017), Schultze & Cumbaa (2017), Müller et al. (2018), Van Viersen & Taghon (2020), and Mondéjar‐Fernández et al. (2021).

**Supplemental References**

**Anderson LI, Poschmann M, Brauckmann C**. **1998**. On the Emsian (Lower Devonian) arthropods of the Rhenish Slate Mountains: 2. The synziphosurine *Willwerathia*. *Paläontologische Zeitschrift* **72** **(3-4)**: 325–336.

**Anderson LI**. **1999**. A new specimen of the Silurian synziphosurine arthropod *Cyamocephalus*. *Proceedings of the Geologists' Association* **110** **(3)**: 211–216.

**Boucot AJ, Janis C**. **1983**. Environment of the early Paleozoic vertebrates. *Palaeogeography, Palaeoclimatology, Palaeoecology* **41** **(3-4)**: 251–287.

**Braddy SJ, Poschmann M, Tetlie OE**. **2008**. Giant claw reveals the largest ever arthropod. *Biology Letters* **4** **(1)**: 106–109.

**Briggs DEG, Clarkson ENK**. **1987**. An enigmatic chordate from the Lower Carboniferous Granton ‘shrimp‐bed’of the Edinburgh district, Scotland. *Lethaia* **20** **(2)**: 107–115.

**Budil P, Manda Š, Tetlie OE**. **2014**. Silurian carcinosomatid eurypterids from the Prague Basin (Czech Republic). *Bulletin of Geosciences* **89** **(2)**:

**Burrow CJ, Turner S, Maisey JG, Desbiens S, Miller RF**. **2017**. Spines of the stem chondrichthyan *Doliodus latispinosus* (Whiteaves) comb. nov. from the Lower Devonian of eastern Canada. *Canadian Journal of Earth Sciences* **54** **(12)**: 1248–1262.

**Chlupáč I, Kříž J, Schönlaub HP, Klapper G, Zikmundová J**. **1980**. Silurian and Devonian conodont localities of the Barrandian. Second European Conodont Symposium (ECOS II), Guidebook, Abstracts: Abhandlungen des Geologisches Bundesaltsalt. p 147–180.

**Chlupáč I**. **1994**. Pterygotid eurypterids (Arthropoda, Chelicerata) in the Silurian and Devonian of Bohemia. *Journal of the Czech Geological Society* **39** **(2-3)**: 147–162.

**Dunlop JA, Poschmann M**. **1997**. On the Emsian (Lower Devonian) arthropods of the Rhenish Schiefergebirge: 1. *Xenarachne*, an enigmatic arachnid from Willwerath, Germany. *Paläontologische Zeitschrift* **71** **(3)**: 231–236.

**Fürsich FT, Hurst JM**. **1980**. Euryhalinity of Palaeozoic articulate brachiopods. *Lethaia* **13** **(4)**: 303–312.

**Fyffe L, Johnson S, van Staal C**. **2016**. A review of Proterozoic to Early Paleozoic lithotectonic terranes in the northeastern Appalachian orogen of New Brunswick, Canada, and their tectonic evolution during Penobscot, Taconic, Salinic, and Acadian orogenesis. *Atlantic Geology* **47**: 211–248.

**Horný RJ**. **2004**. *Kosovina*, a new Silurian tryblidiid genus (Mollusca, Tergomya) from Bohemia (Czech Republic). *Acta Musei Nationalis Pragae, Series B, Natural History* **60** **(3-4)**: 143–148.

**Horný RJ**. **2005**. Muscle scars, systematics and mode of life of the Silurian family Drahomiridae (Mollusca, Tergomya). *Acta Musei Nationalis Pragae, Series B, Historia Naturalis* **61** **(1-2)**: 53–76.

**Howells Y**. **1982**. Scottish Silurian trilobites. *Monograph of the Palaeontographical Society* **135**: 1–70.

**Jones TR, Woodward H**. **1885**. Notes on the British species of *Ceratiocaris*. *Geological Magazine* **2** **(9)**: 385–397.

**Kennedy KL, Miller RF, Gibling MR**. **2012**. Palaeoenvironments of Early Devonian fish and other aquatic fauna of the Campbellton Formation, New Brunswick, Canada. *Palaeogeography, Palaeoclimatology, Palaeoecology* **361**: 61–72.

**Kříž J, Jaeger H, Paris F, Schönlaub HP**. **1986**. Pridoli-the Fourth Subdivison of the Silurian. *Jahrbuch der Geologischen Bundesanstalt* **129**: 291–360.

**Kříž J**. **1992**. *Silurian field excursions: Prague Basin (Barrandian), Bohemia*. Cardiff: National Museum of Wales.

**Manda Š, Turek V**. **2009**. A Silurian oncocerid with preserved colour pattern and muscle scars (Nautiloidea). *Bulletin of Geosciences* **84** **(4)**: 755–766.

**Manda Š, Frýda J**. **2010**. Silurian-Devonian boundary events and their influence on cephalopod evolution: evolutionary significance of cephalopod egg size during mass extinctions. *Bulletin of Geosciences* **85** **(3)**: 513–540.

**Märss T, Ritchie A**. **1997**. Articulated thelodonts (Agnatha) of Scotland. *Earth and Environmental Science Transactions of the Royal Society of Edinburgh* **88** **(3)**: 143–195.

**Mergl M**. **2001**. Lingulate brachiopods of the Silurian and Devonian of the Barrandian (Bohemia, Czech Republic). *Acta Musei Nationalis Pragae, Series B – Historia Naturalis* **27**: 1–49.

**Mergl M**. **2019**. Lingulate brachiopods across the Kačák Event and Eifelian-Givetian boundary in the Barrandian area, Czech Republic. *Bulletin of Geosciences* **94** **(2)**: 169–186.

**Miller RF, Kennedy K, Gibling MR**. **2012**. A eurypterid from the lacustrine facies of the Early Devonian Campbellton Formation, New Brunswick, Canada. *Atlantic Geology* **48**: 14–19.

**Mondéjar‐Fernández J, Friedman M, Giles S**. **2021**. Redescription of the cranial skeleton of the Early Devonian (Emsian) sarcopterygian *Durialepis edentatus* Otto (Dipnomorpha, Porolepiformes). *Papers in Palaeontology* **7** **(2)**: 789–806.

**Müller P, Hahn G, Franke C, Thuy B**. **2018**. A new paedomorphic protasterid brittle star (Echinodermata, Ophiuroidea) from the Early Devonian of Luxembourg and Germany. *Swiss Journal of Palaeontology* **137** **(2)**: 327–335.

**Perrier V, Siveter DJ, Williams M, Palmer D**. **2019**. British Silurian myodocope ostracods. *Monographs of the Palaeontographical Society* **172** **(651)**: 1–64.

**Poschmann M**. **2006**. The eurypterid *Adelophthalmus sievertsi* (Chelicerata: Eurypterida) from the Lower Devonian (Emsian) Klerf Formation of Willwerath, Germany. *Palaeontology* **49** **(1)**: 67–82.

**Poschmann M, Franke C**. **2006**. Arthropods and trace fossils from the Lower Devonian (Emsian) of the West Eifel region/Germany and the Grand Duchy of Luxembourg. *Ferrantia* **46**: 97–115.

**Poschmann M, Tetlie OE**. **2006**. On the Emsian (Lower Devonian) arthropods of the Rhenish Slate Mountains: 5. Rare and poorly known eurypterids from Willwerath, Germany. *Paläontologische Zeitschrift* **80** **(4)**: 325–343.

**Poschmann M, Bergmann A, Kühl G**. **2017**. First record of eurypterids (Chelicerata, Eurypterida) from the Lower Devonian (Lower Emsian) Hunsrück Slate (SW Germany). *PalZ* **91** **(2)**: 163–169.

**Rolfe WDI, Fritz MA**. **1966**. Recent evidence for the age of the Hagshaw Hills Silurian Inlier, Lanarkshire. *Scottish Journal of Geology* **2** **(2)**: 159–164.

**Schultze H-P, Cumbaa SL**. **2017**. A new Early Devonian (Emsian) arthrodire from the Northwest Territories, Canada, and its significance for paleogeographic reconstruction. *Canadian Journal of Earth Sciences* **54** **(5)**: 461–476.

**Shear WA, Gensel PG, Jeram AJ**. **1996**. Fossils of large terrestrial arthropods from the Lower Devonian of Canada. *Nature* **384** **(6609)**: 555–557.

**Südkamp WH, Burrow CJ**. **2007**. The acanthodian *Machaeracanthus* from the Lower Devonian Hunsrück Slate of the Hunsrück region (Germany). *Paläontologische Zeitschrift* **81** **(1)**: 97–104.

**Tetlie OE, Braddy SJ**. **2003**. The first Silurian chasmataspid, *Loganamaraspis dunlopi* gen. et sp. nov. (Chelicerata: Chasmataspidida) from Lesmahagow, Scotland, and its implications for eurypterid phylogeny. *Earth and Environmental Science Transactions of The Royal Society of Edinburgh* **94** **(3)**: 227–234.

**Tetlie OE, Poschmann M**. **2008**. Phylogeny and palaeoecology of the Adelophthalmoidea (Arthropoda; Chelicerata; Eurypterida). *Journal of Systematic Palaeontology* **6** **(2)**: 237–249.

**Van Viersen AP, Taghon P**. **2020**. A poorly diversified trilobite association from the lower Emsian (Lower Devonian) in the Sankt Vith area (East Belgium). *Geologica Belgica* **23**: 19–28.

**Wehrmann A, Hertweck G, Brocke R, Jansen U, Königshof P, Plodowski G, Schindler E, Wilde V, Blieck A, Schultka S**. **2005**. Paleoenvironment of an Early Devonian land–sea transition: a case study from the southern margin of the Old Red Continent (Mosel Valley, Germany). *Palaios* **20** **(2)**: 101–120.

**Wilson HM**. **2006**. Juliformian millipedes from the Lower Devonian of Euramerica: implications for the timing of millipede cladogenesis in the Paleozoic. *Journal of Paleontology* **80** **(4)**: 638–649.
